# “Sponge pattern” of the spleen: a rarely described high-frequency ultrasound pattern in HIV-positive patients

**DOI:** 10.1186/s13089-022-00297-z

**Published:** 2023-02-03

**Authors:** Tom Heller, Francesco Taccari, Kelvin Rambiki, Tapiwa Kumwenda, Enrico Brunetti, Claudia Wallrauch

**Affiliations:** 1 Lighthouse Clinic, Kamuzu Central Hospital, Area 33, Mzimba Street, P.O. Box 106, Lilongwe, Malawi; 2 International Training and Education Center for Health, University of Washington, Seattle, WA USA; 3 Dipartimento di Scienze di Laboratorio e Infettivologiche, Fondazione Policlinico Universitario A. Gemelli IRCCS, Rome, Italy; 4 Division of Infectious and Tropical Diseases, University of Pavia, IRCCS S. Matteo Hospital Foundation, Pavia, Italy

**Keywords:** High-frequency, Ultrasound, Spleen, HIV, HHV-8

## Abstract

**Background:**

The spleen is frequently scanned in workup of infections. Hypoechoic splenic micro-abscesses are known signs of disseminated tuberculosis in HIV co-infected patients. The spleen of HIV patients is thus often scanned using high-frequency transducers.

**Methods and findings:**

We describe a reticulo-nodular “sponge pattern” in the spleen of an HIV-positive patient with Hodgkin’s lymphoma. Disseminated throughout the spleen, very small (1.5–2.0 mm) hypoechoic lesions having a branching reticulo-nodular distribution were seen. The lesions partly, but not entirely, follow splenic vasculature. Review of stored images of other patients identified 15 more cases showing a similar pattern. All patients were HIV positive, almost all with CD4 counts below 200 cells/mm^3^. Seven (44%) were additionally diagnosed with HHV-8-associated diseases, but the pattern was seen with various underlying opportunistic infections.

**Discussion and conclusion:**

After comparison with spleen microscopic anatomy, we hypothesize that the white pulp of spleens in our patients is hyperplastic or otherwise changed in consistency to be better visible by high-frequency ultrasound. Concomitant human herpesvirus-8 infection may be another cause of this visible white pulp. While we can only speculate about the etiology of the splenic “sponge pattern,” it needs to be recognized as it may be misinterpreted as splenic micro-abscesses of disseminated infections, like tuberculosis in severely immune-compromised patients.

**Supplementary Information:**

The online version contains supplementary material available at 10.1186/s13089-022-00297-z.

## Introduction

The spleen as large lymphatic organ is often assessed by ultrasound in the workup of infectious diseases; homogeneous splenomegaly is a very common finding in disseminated tropical and non-tropical infections. Over the past decade, focal micro-abscesses in the spleen as sign of disseminated TB in HIV co-infected patients have been described [1]. The lesions are hypoechoic and vary in size from few millimeters to one centimeter and have been found to be significantly associated with the diagnosis of disseminated TB in African settings [2, 3]. They are thus included in focused screening protocols [4, 5]. As the spleen lesions disseminated throughout the parenchyma and the organ is in the trans-costal view only few centimeters under the skin (especially in lean patients with loss of weight), high-frequency ultrasound transducers are frequently used as the micro-abscesses are clearer visible. This increased focus on the sonographic micro-anatomy of the spleen may lead to the detection of other patterns in the organ, which need to be known to avoid misinterpretation and wrong diagnoses.

Here we describe a case of a reticulo-nodular “sponge pattern” in the spleen of an HIV-positive patient with Hodgkin’s lymphoma. Infectious disease colleagues frequently using ultrasound were consulted to report individual cases with similar patterns. Finally, the sonographic pattern was compared to low-magnification spleen tissue slides in our pathology department.

## Case vignette

A 14-year-old HIV-positive girl was seen in Lighthouse clinic, a referral-level HIV clinic in Lilongwe, Malawi. She reported weight loss, abdominal pain, and inguinal lymph node swellings. The patient was prescribed antiretroviral therapy for more than 6 years but was virologically not suppressed due to poor adherence. CD4 count was 145 cells/ml; a cryptococcal antigen in serum and a urine LAM test for disseminated TB were both negative.

As ultrasound is frequently performed in our clinic to screen for signs of extrapulmonary TB [6], a scan was performed using a convex 3.5 MHz and a linear 7.5 MHz transducer (Mindray DC-30, China). Multiple intra-abdominal and inguinal enlarged lymph nodes (> 2 cm diameter) were seen. In the normal-sized spleen, numerous hypoechoic lesions of approx. 1–2 mm diameter were detected. Assuming hematological malignancy and disseminated TB as the most likely diagnoses, an ultrasound-guided lymph node biopsy was performed. PCR for *Mycobacterium tuberculosis* (GeneXpert MTB/RIF, Cepheid, USA) of the lymph node material was negative; histology confirmed Hodgkin’s lymphoma and the patient was referred for chemotherapy to the pediatric oncology department.

## Description of the ultrasound findings and analysis

**Fig. 1:**
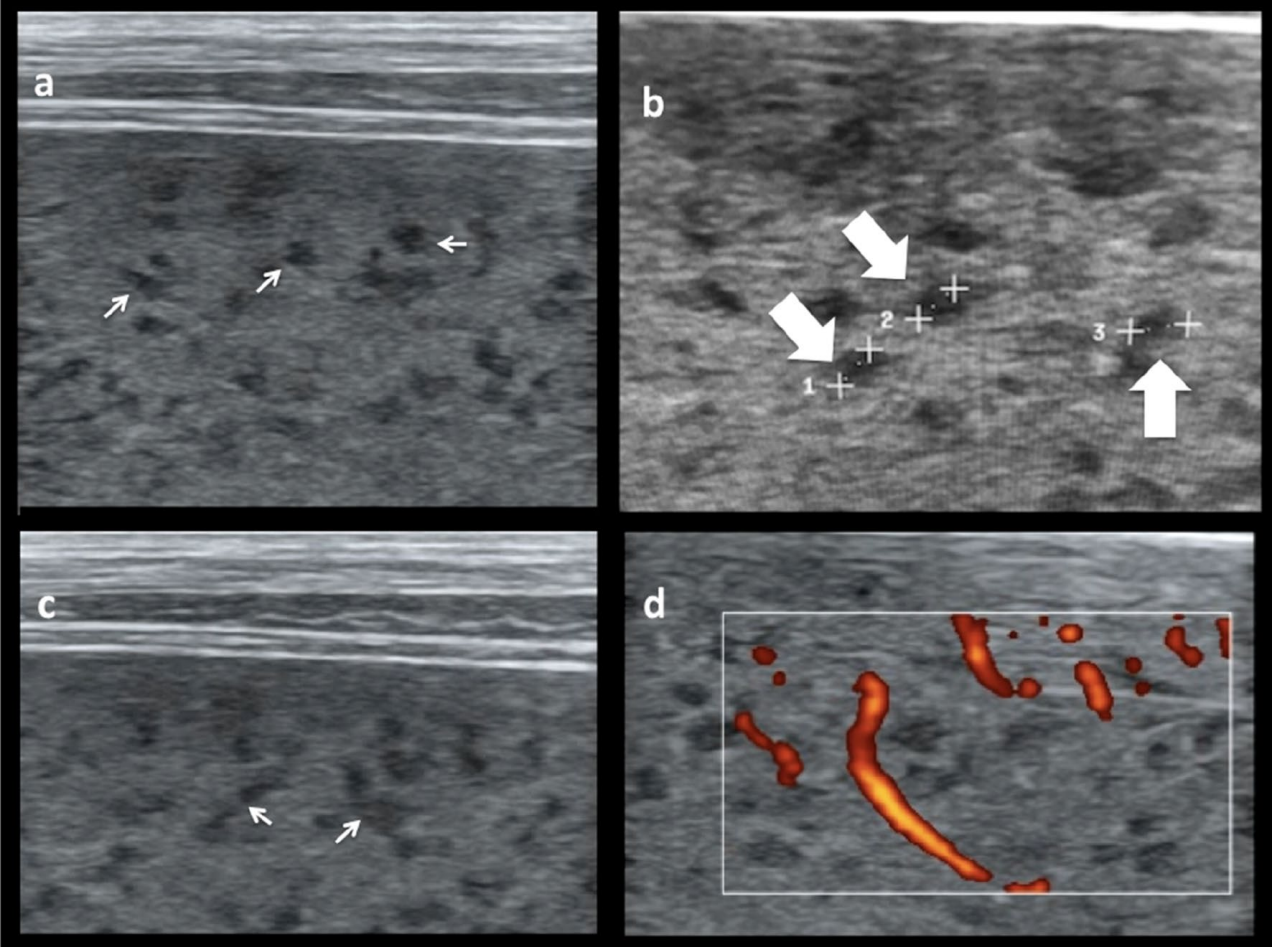
Sonographic appearance of the spleen of index patient. **a** Numerous tiny hypoechoic areas (arrows) are seen throughout the splenic tissue. **b** The areas measure about 2 mm in diameter (Block arrows: 1: 1.9 mm, 2: 2.0 mm, 3: 2.4 mm). **c** In some still views a more linear appearance is noted which is better visible in the moving scan (see Additional file 1: Video clip S1a). **d** Using the power mode it can be seen that some of the structures follow the vasculature (see Additional file 2: Video clip S1b); others seem isolated

The detected lesions in our patient were disseminated throughout the sonographically visible spleen tissue (Fig. 1). The lesions were about 1.5–2.0 mm in diameter and the distance between lesions varied from 1.5 to 4 mm. Using the movement of the transducer, lesions appeared not isolated but seemed to have a branching, reticulo-nodular distribution throughout the parenchyma (Additional file 1: Video clip S1a). Also in some of the still views, the lesions gave a slightly linear appearance. Using power Doppler, images reveal that the lesions partly follow vasculature of the spleen, but not all of them are connected (Additional file 2: Video clip S1b). “Sponge pattern” was coined as a descriptive term.

**Fig. 2:**
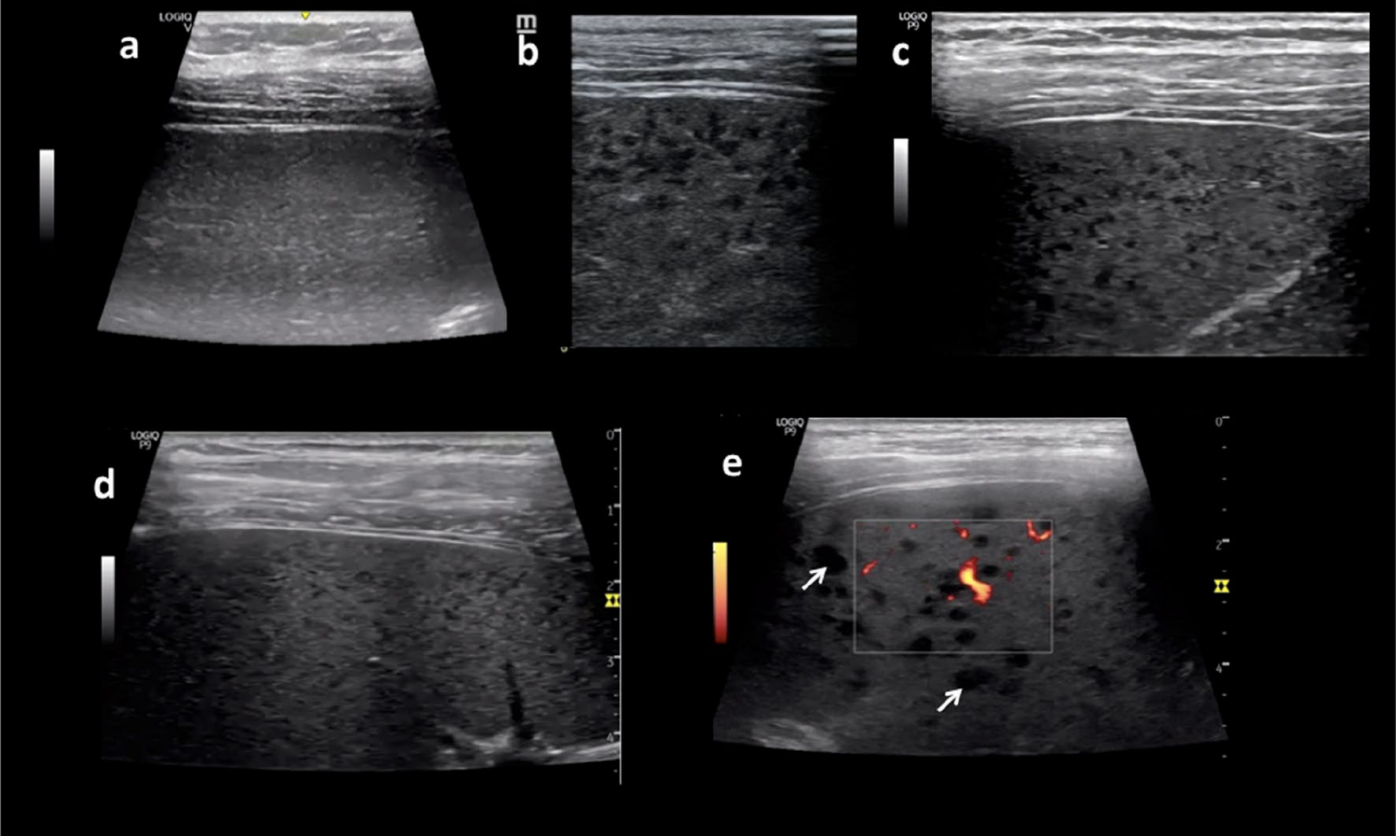
Examples of spleen tissue **a** Normal homogenous spleen tissue; **b** Sponge pattern in a patient with disseminated Kaposi’s sarcoma, **c** with MAC infection (also in Additional file 3: Video clip S1a) **d** with underlying pulmonary PCP infection. **e** Micro-abscesses in the spleen of patient with disseminated TB for comparison

The description of the pattern was shared with colleagues frequently doing clinical ultrasound in patients with infectious diseases. In total, spleens of 16 patients showing a similar reticulo-nodular pattern were identified. Patient data are summarized in Table 1. All patients were HIV positive and 93% of patients with available CD4 counts had less than 200 cells/mm, hence fulfilling the definition of advanced HIV disease. Seven of the patients (44%) had additionally human herpes virus-8 (HHV-8)–associated diseases (six Kaposi’s sarcoma, one Multicentric Castleman Disease). Besides the Hodgkin’s lymphoma of the index patient, pneumocystis pneumonia, pulmonary NTM infection, pneumococcal severe bloodstream infection, and disseminated MAC infection were diagnosed in individual cases. The remaining four cases had newly found HIV infections without diagnosis of further systemic opportunistic infection. Sample images of the cases are provided in Fig. 2 (and in Additional file 3: Video clip S2) contrasting the changes with images of normal spleen texture and of tuberculous micro-abscesses.

**Table 1:** Characteristics of patients identified with reticulo-nodular “sponge pattern” of the spleen

Patient	Age	Sex	HIV status	Opportunistic infection/disease	Origin
1	14	F	+ (CD4 145)	Hodgkin’s lymphoma	Malawi
2	16	F	+ (CD4 n.a.)	Pneumococcal septicemia	Malawi
3	34	M	+ (CD4 54)	KS	Malawi
4	26	M	+ (CD4 132)	KS	Malawi
5	46	M	+ (CD4 103)	KS	Malawi
6	34	F	+ (CD4 124)	KS	Malawi
7	42	F	+ (CD4 11)	PCP	Ukraine
8	27	M	+ (CD4 193)	KS	Italy
9	47	F	+ (CD4 25)	Disseminated MAC	Italy
10	29	F	+ (CD4 170)	Pulmonary MAC	Nigeria
11	31	M	+ (CD4 68)	HIV infection only (+ oral thrush)	Brazil
12	30	F	+ (CD4 269)	HIV infection only	Colombia
13	42	M	+ (CD4 190)	HIV infection only	Italy
14	40	M	+ (CD4 13)	KS	Italy
15	16	F	+ (CD4 27)	HIV infection only	Malawi
16	43	M	+ (CD4 92)	Multicentric Castleman Disease	Italy

**Fig. 3:**
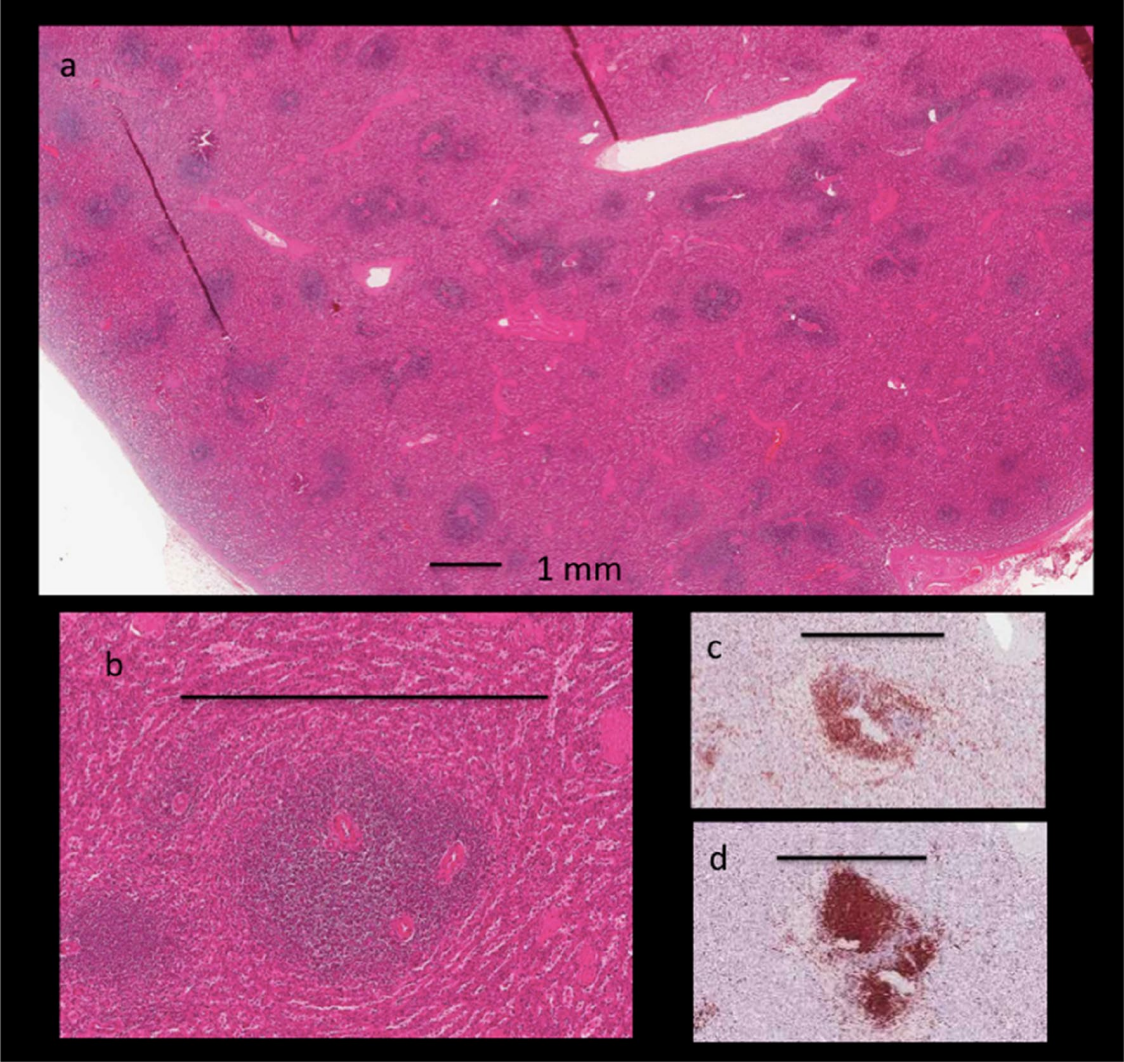
Microscopic anatomy of the spleen (unrelated patient without infection, splenectomy due to injury) **a** Disseminated darker lymphatic follicle and periarterial lymphatic sheaths (PALS) (= ”white pulp”) are embedded in the surrounding erythrocyte rich “red pulp” (H&E stain). **b** Enlarged area of PALS (H&E stain). **c** B-cells (CD20 +) located more peripheral and d) T-cells (CD3 +) closer to the arteriole in the PALS. All size bars = 1 mm

Pathological specimens of unrelated extirpated spleens in the specimen bank of the UNC pathology project at Kamuzu Central Hospital, Lilongwe were reviewed for comparison (Fig. 3). The sonographic findings seem to mirror the anatomical distribution of the white pulp, the lymphocyte-rich areas within the spleen. This lymphatic tissue is distributed along the arteries and has a branching appearance. Their central areas, the periarterial lymphatic sheaths (PALS), are rich in T-lymphocytes. Lymphatic follicles, predominantly B-cell regions, are associated with the PALS but also dispersed individually throughout the spleen. The size of normal white pulp areas is around 1 mm in diameter. Overall the size and distribution resembles the sonographic images seen in our HIV-positive patients.

## Discussion

The spleen is the largest lymphoid organ and the largest filter for blood in the body. Spleen parenchyma is divided into two functionally and morphologically distinct compartments (red and white pulp). Red pulp forms the majority of spleen tissue and consists of blood filled splenic sinusoids. White pulp is mainly made of PALS and lymphoid follicles. The PALS consists of a central artery surrounded by a sheath of lymphocyte-rich tissue. Its inner layer mainly consists of T-lymphocytes, while the outer layer shows a more diverse cellular picture, containing T- and B-lymphocytes. Lymphoid follicles of the spleen contain mainly B-lymphocytes. Splenic follicles demonstrate prominent marginal zones; here marginal zone macrophages expressing C-type lectins and a scavenger-receptor binding antigens on pathogens, such as Mycobacterium tuberculosis and Streptococcus pneumoniae, are located [7].

We hypothesize that the white pulp in our patients may be hyperplastic or otherwise changed in its consistency to be able to be seen more clearly in high-frequency sonography. A variety of pathogens can stimulate white pulp and cause reactive lymphoid hyperplasia [8]. Follicular hyperplasia often with marginal zone hyperplasia is seen in autoimmune diseases but also as reactive process in the setting of known lymphoma [8]. Generalized viral infections, especially EBV but also CMV and herpes virus infection can cause reactive lymphoid hyperplasia without germinal center formation in the spleen. In these spleens, expansion of the red pulp with partial loss of the white pulp is the main histological finding [8]. Also HIV infection is known to change the architecture of the spleen. Autopsy studies showed white pulp depletion and also perivascular hyaline fibrosis, possibly as an exaggeration of normal splenic histology, especially in advanced HIV disease stages [9]. This accentuation could lead to better visibility on ultrasound.

A possible explanation of the visible pulp could be concomitant HHV-8 infection, supported by the fact that almost half of the patients had overt opportunistic disease due to HHV-8. HHV-8 is the causal agent of KS but is also pathogenetically related to several lymphoproliferative disorders, including Multicentric Castleman Disease [10]. These diseases were diagnosed frequently, whether the remaining patients were infected sub-clinically with HHV-8 cannot be ascertained. It can nevertheless be speculated that HHV-8, possibly in conjunction with the advanced HIV disease, may cause changes of white pulp in the spleen.

The possibility to visualize the white pulp as a reticulo-nodular pattern has been described previously in pediatric patients using a 13 MHz linear array transducer [11]. A prominent reticulo-nodular pattern similar to the one seen in our patients was seen in 11 of 100 children; the pattern was associated with age and most frequently seen in the older children but not beyond the age of 10 years. The authors did not find an association with sex or BMI of the patients, but suggest that the increasing size of children makes the access for high-frequency sonography less feasible in larger patients. To our knowledge similar findings have not been described in adults.

Ultrasound evaluation of the spleen in patients with HIV and symptoms suggestive of TB in endemic regions is a viable diagnostic adjunct and detection of splenic micro-abscesses is probably sufficient indication to initiate TB treatment [3]. Nevertheless, in recent years a variety of disseminated infections have been shown to cause similar changes in the spleen; clinical, geographical and epidemiological information must therefore be considered in the diagnosis. Disseminated bartonellosis [12] and disseminated MAC infection [13] can cause splenic micro-abscesses in HIV patients. In Southeast Asian patients, melioidosis of the spleen must be considered as a differential diagnosis [14]. In areas where brucellosis is endemic, this disease poses another potential cofounder [15]. Leishmaniasis causes predominantly homogeneous splenomegaly but focal lesions are as well described [16].

The sonographic pattern described in this article adds another potential confounder because the “sponge pattern” may be interpreted as disseminated micro-abscesses in the spleen. Nevertheless, the lesions are smaller than TB micro-abscesses and the branching sponge-like appearance can be differentiated when moving the transducer. About the etiology of the pattern, not described in adult patients, can only be speculated, but it seems associated with advanced HIV infection and possibly with HHV-8 infection. Further studies of the pattern and its possible associated diseases are needed and clinical observations from other sonographers required.

## Supplementary Information

**Additional file 1: Video clip S1a:** Spleen of HIV + patient with Hodgkin’s lymphoma (Linear 7.5 MHz transducer, Mindray DC-30). The 1–2 mm diameter, hypoechoic lesions are seen throughout the visible spleen. They partially have a linear and branching appearance.

**Additional file 2: Video clip S1b:** Spleen of HIV + patient with Hodgkin’s lymphoma (Linear 7.5 MHz transducer with power Doppler, Mindray DC-30). A partial association of the hypoechoic areas with the vessel can be appreciated.

**Additional file 3: Video clip S2:** Spleen of HIV + patient with MAC infection showing a similar pattern (Linear 10 MHz transducer, GE Logic P9).

## Data Availability

Not applicable.
